# A Mechanism Linking Id2-TGFβ Crosstalk to Reversible Adaptive Plasticity in Neuroblastoma

**DOI:** 10.1371/journal.pone.0083521

**Published:** 2013-12-23

**Authors:** Lina Chakrabarti, Bi-Dar Wang, Norman H. Lee, Anthony D. Sandler

**Affiliations:** 1 The Joseph E. Robert Center for Surgical Care, Children’s National Medical Center, Washington, D.C., United States of America; 2 The Sheikh Zayed Institute for Pediatric Surgical Innovation, Children’s National Medical Center, Washington, D.C., United States of America; 3 Department of Pharmacology and Physiology, George Washington University Medical Center, Washington, D.C., United States of America; University of Kentucky College of Medicine, United States of America

## Abstract

The ability of high-risk neuroblastoma to survive unfavorable growth conditions and multimodal therapy has produced an elusive childhood cancer with remarkably poor prognosis. A novel phenomenon enabling neuroblastoma to survive selection pressure is its capacity for reversible adaptive plasticity. This plasticity allows cells to transition between highly proliferative anchorage dependent (AD) and slow growing, anoikis-resistant anchorage independent (AI) phenotypes. Both phenotypes are present in established mouse and human tumors. The differential gene expression profile of the two cellular phenotypes in the mouse Neuro2a cell line delineated pathways of proliferation in AD cells or tyrosine kinase activation/ apoptosis inhibition in AI cells. A 20 fold overexpression of inhibitor of differentiation 2 (Id2) was identified in AD cells while up-regulation of genes involved in anoikis resistance like PI3K/Akt, Erk, Bcl2 and integrins was observed in AI cells. Similarly, differential expression of Id2 and other genes of interest were also observed in the AD and AI phenotypes of human neuroblastoma cell lines, SK-N-SH and IMR-32; as well as in primary human tumor specimens. Forced down-regulation of Id2 in AD cells or overexpression in AI cells induced the cells to gain characteristics of the other phenotype. Id2 binds both TGFβ and Smad2/3 and appears critical for maintaining the proliferative phenotype at least partially through negative regulation of the TGFβ/Smad pathway. Simultaneously targeting the differential molecular pathways governing reversible adaptive plasticity resulted in 50% cure of microscopic disease and delayed tumor growth in established mouse neuroblastoma tumors. We present a mechanism that accounts for reversible adaptive plasticity and a molecular basis for combined targeted therapies in neuroblastoma.

## Introduction

Neuroblastoma is a pediatric solid tumor originating from neural crest progenitors. This disease displays considerable clinical variability, reflected in patient outcomes that range from spontaneous regression to lethal disease [1], [2], [3]. Moreover, neuroblastoma exhibits a wide range of differentiated phenotypes, from undifferentiated tumors to tumors containing a neural crest-derived differentiated cell state [4]. Heterogeneity within cancer cell populations is common, in which many tumors contain phenotypically and functionally different cancer cell populations [5], [6], [7]. Tumor heterogeneity can arise through multiple mechanisms including genetic/epigenetic changes [8], [9], microenvironmental pressure [10], [11], anoikis resistance [12], [13], [14], [15] and cancer stem cell populations [16], [17], [18].

Tumor cell adaptation is an important phenomenon as it could enable tumors to evade immune surveillance, survive unfavorable conditions or escape radio- or chemotherapy. We have recently described a novel form of adaptive cell transformation, termed reversible adaptive plasticity and demonstrated that neuroblastoma cells are plastic, dynamic and optimize their ability to survive by switching their phenotype [19]. We identified two defined neuroblastoma phenotypes with anchorage dependent (AD) and anchorage independent (AI) growth patterns in mouse and human cell lines under distinct culture conditions [19]. Since neuroblastoma tumor cells arise from embryonic neural crest cells, the AI cells are grown as spheroids in neural stem cell serum free culture conditions while the AD counterparts proliferate rapidly and attach to the plate in regular serum rich media [19]. The importance of this finding translates to tumor growth as both phenotypes are capable of reversible transition and specific molecular markers enabled us to observe both cell types in established mouse and human neuroblastoma tumors. We have also identified this phenomenon in multiple other tumor types (unpublished observations) and indeed, other human cancers frequently display substantial intra-tumor heterogeneity in cellular morphology and gene expression [5].

Resistance to anoikis is another molecular mechanism that could afford tumors aggressive, chemo-resistant properties. Anoikis is the induction of apoptosis induced by loss of cell adhesion. Overcoming anoikis and adapting an anchorage independent state is a crucial step in malignant transformation and metastasis, making anoikis resistance a natural pre-requisite for aggressive growth of cancer [14]. Tumor cells have developed a variety of strategies to avoid anoikis, by altering integrin expression in both squamous carcinoma [20] and melanoma cells [21] and by activation of the integrin/FAK/PI3K pathway in murine lung carcinoma cells [22]. Resistance to anoikis occasionally is mediated by oxidative stress, in which reactive oxygen species activate the Src tyrosine kinase that in turn activates the pro-survival pathways contributing to survival [23]. Furthermore, aberrant activation of Src and the proto-oncogenes Ras/Raf are reported to confer resistance to anoikis through PI3K/Akt signaling in various cancers [24], [25], [26]. Normally, cells grown *in vitro* under conditions of loss of cell-matrix anchorage and serum starvation undergo anoikis. However, in our recent study we observed that the majority of Neuro2a cells growing under these conditions overcame apoptosis and survived as the AI phenotype [19]. Therefore, we reasoned that addition of growth factors in the culture medium may have activated growth factor receptors and other anti-apoptotic and survival pathways contributing to anoikis resistance of surviving AI cells. Since it is now widely accepted that tumor cells optimize their ability to survive anoikis by over-activation of survival/proliferation cascades, we assessed these pathways in the AI phenotypes of the neuroblastoma cells.

In an effort to investigate the mechanism(s) driving reversible phenotypic transition in neuroblastoma, the gene expression profile of the AD and AI phenotypes of a mouse neuroblastoma cell line was studied. Gene array analysis of Neuro2a cells revealed remarkable differences between the two phenotypes *in vitro* and elucidated the molecular patterns and pathways associated with each phenotype. In particular, Id2 was 20 fold overexpressed in AD cells compared to AI cells. In addition, a wide array of genes governing anoikis resistance was overexpressed in the AI cells. Id2 was also found to be expressed in the AD phenotype of human cell lines as well as in primary human tumor specimens and many of the other survival/proliferation genes of interest were also differentially expressed in human neuroblastoma AD and AI cell phenotypes. In view of Id2’s function as an effector of n-myc, its role in cell proliferation and its remarkably high differential expression in cell phenotypes, we hypothesized that Id2 plays a critical role in maintaining the AD phenotype. Either transient or stable knock-down of Id2 in AD cells and forced overexpression in AI cells resulted in the cells adapting characteristics of the other phenotype and confirmed the key role of Id2 in reversible adaptive plasticity of neuroblastoma cells. Furthermore, our results indicate that Id2 functions at least partially through negative regulation of the TGFβ/Smad pathway as Id2 binds both TGFβ and Smad2/3 and suppression of Id2 in AD cells activated Smad signaling and phenotypic transition to an AI-like state. Finally, tumor growth in a mouse neuroblastoma model was remarkably suppressed by therapeutically targeting the molecular pathways governing reversible adaptive plasticity.

## Materials and Methods

### Animals

Female A/J mice (6 weeks old) were purchased from Jackson Laboratory (Bar Harbor, ME). The animals were acclimated for 4–5 days prior to tumor challenge. All procedures involving animals were approved by the Institutional Animal Care and Use Committee of Children’s National Medical Center (CNMC), Washington DC.

### Human tumor specimens

De-identified human neuroblastoma samples were obtained from the Pathology department of CNMC. Written informed consents were obtained from the parents or guardians of the patients in accordance with the Declaration of Helsinki. All procedures involving the use of human tumor specimens were approved by the Institutional Review Board of CNMC.

### Cell lines

Neuro2a is the murine neuroblastoma cell line derived from AJ mice. IMR-32 and SK-N-SH are human cell lines derived from MYCN amplified and MYCN non-amplified neuroblastoma tumors respectively. All cell lines were purchased from ATCC (Manassas, VA) and cultured as previously described [19]. Briefly, AD cells were grown in DMEM (Gibco, Carlsbad, CA) containing 10% fetal bovine serum (FBS, Gibco), 0.5% penicillin/streptomycin (Sigma, St. Louis, MO) and 10% L-glutamine (Sigma). Anchorage independent tumorspheres (AI) from the Neuro2a cells were grown in NeuroCult complete media consisting of NeuroCult Neural Stem Cell (NSC) Basal medium, 1/10 NeuroCult NSC Proliferation supplements, 20 ng/ml EGF, 10 ng/ml bFGF and 2 µg/ml Heparin. NeuroCult media, supplements and growth factors were all purchased from Stem Cell Technologies (Vancouver, BC, Canada).

### Affymetrix gene array

RNA was extracted from the harvested AD and AI Neuro2a cells using the RNeasy kit (Qiagen, Frederick, MD) and gene array was performed using Affymetrix ST mouse GeneChips. Four replicate hybridizations per phenotype were performed. Partek Genomic Suite 6.6 and GeneSpring 12.5 were used to analyze Affymetrix microarray data. Three different methods of data normalization were used (GCRMA-quantile, RMA16, and PLIER16) in which the differential gene expression pattern was compared among the different methods and overlapping genes were considered for further validation. Two-way hierarchical clustering analysis (average linkage algorithm and Euclidean distance metric) was performed by using_Partek Genomic Suite 6.6. Ingenuity Pathway Analysis was used and power calculations indicate discrimination of 1.5-fold expression changes at α = 0.05 with >90% power.

### Cell transfection

The AD phenotype of Neuro2a, SK-N-SH and IMR-32 cells was transfected with either mouse or human specific Id2-siRNA (Santa Cruz Biotechnology, Santa Cruz, CA) to down regulate Id2. Both the siRNAs are a pool of 3 target-specific 19–25 nucleotide siRNAs. In a separate assay the Neuro2a AD cells were transfected with anti-sense oligonucleotides complementary to Id2 (Id2-AS, 5′-AGGCTTTCATGCTGACCGC-3′) (IDT, Iowa City, IA) to down regulate Id2. Control-siRNA or mismatched oligonucleotide (Id2-msm, 5′-GCGAGTTGTCGCACGGTCT-3′) was used as control [27]. To overexpress Id2, the AI phenotype of Neuro2a cells was transiently transfected with Id2-IRES-GFP plasmid (GenScript, Piscataway, NJ). IRES-GFP was used as control plasmid. The siRNA (Id2 or control), oligos (AS or msm) or the plasmids were mixed with Lipofectamine 2000 (Invitrogen, Grand Island, NY) and added to the cells for 8 hours. Following transfection, the cells were harvested after 24 hours and analyzed for proliferation, cell cycle, apoptosis and Western blotting.

### Stable cell transfection

The AD phenotype of Neuro2a cells were transduced with Id2-shRNA lentiviral particles (Santa Cruz Biotechnology) for stable knockdown of Id2. The stable clones expressing the Id2-shRNA were selected using Puromycin according to the manufacturer’s instructions. Scrambled shRNA lentiviral particles were used as control. The transduced Id2 knock down cells were plated on NeuroCult complete media (AI conditioning) and monitored for sphere forming potential to examine their propensity for phenotypic transition.

### Cell proliferation assay

5-bromodeoxyuridine (BrdU, 10 µM) was added to the cell culture medium two hours prior to cell harvest. The incorporated BrdU was stained with BrdU-APC antibody using the BrdU Flow kit (BD Biosciences, San Jose, CA) according to the manufacturer’s protocol and measured by flow cytometry using FACSCalibur (BD Biosciences). Analysis was done by FlowJo software (Tree Star, Inc., Ashland, OR).

### Cell cycle analysis

The AD and AI cells were harvested from culture medium, washed in cold phosphate buffered saline (PBS) and fixed in 70% ethanol at –20°C for 2 hours. Cells were centrifuged, washed with cold PBS and resuspended in propidium iodide mix (40 µg/ml PI and 0.1 mg/ml RNase in PBS). After 30 minutes incubation at 37°C, cells were acquired in FACSCalibur and analysis was done by FlowJo software.

### Apoptosis assay

The AD and AI cells were stained with FITC-conjugated AnnexinV (BD Biosciences) for 20 minutes followed by 7-AAD for 5 minutes. Cells were analyzed immediately for AnnexinV/7-AAD expression using FACSCalibur. Analysis was done by FlowJo software.

### TGFβ inhibition in vitro

The function of the endogenous TGFβ pathway in Neuro2a AD cells after Id2 down regulation was inhibited by TGFβ neutralizing antibody (1D11, R&D Systems, Minneapolis, MN) or TGFβ type I/II receptor inhibitor (LY2109761, Selleck Chemicals, Houston, TX) in separate assays. Together with Id2-siRNA, 10 µg/ml of 1D11 or 5 µM LY2109761 was added to the cells. Twenty-four hours later apoptosis assay was performed and cell lysates were prepared for Western blot analysis.

### In vivo tumor growth and drug treatments

Mice were injected subcutaneously with 1×10^6^ Neuro2a cells of the AD phenotype and divided into 5 groups as follows, Group 1: treatment with vehicles only, Group 2: treatment with doxorubicin (Sigma, St. Louis, MO) and metformin (Sigma), Group 3: treatment with sorafenib (Selleck Chemicals, Houston, TX), Group 4: treatment with doxorubicin, metformin and sorafenib and Group 5: treatment with doxorubicin, metformin, sorafenib and LY2109761. All treatments were started on post-inoculation day one and continued for three weeks except for Group 5 which received only two weeks of treatment. Doxorubicin (2.5 mg/Kg, dissolved in water) was administered twice a week intraperitonealy (i.p.), metformin (200 mg/L) was given daily in drinking water, sorafenib (50 mg/Kg, dissolved in oral vehicle consisting of 1% sodium carboxymethylcellulose, 0.5% sodium lauryl sulfate and 0.05% antifoam) was administered daily by oral gavage and LY2109761 (50 mg/Kg, dissolved in oral vehicle) was administered orally twice daily. Tumor growth was monitored on alternate days. Weight loss, lethargy and sick mouse postures with ruffled fur and piloerection were used as indicators of toxicity. The Group 5 treatment strategy was repeated on an established tumor model where drug treatment was started when the tumor was visible and measured about 5 mm in diameter.

### Immunofluorescence assay

Ten micron frozen sections of mouse and human primary tumors were stained for Id2 using rabbit anti-Id2 (Santa Cruz Biotechnology) antibody diluted 1∶250 in PBS containing 0.3% TritonX-100. AlexaFluor 488 conjugated goat anti-rabbit IgG (1∶200, Invitrogen) was used as secondary antibody and DAPI was used as nuclear stain. To determine the background fluorescence, only secondary antibody was used. The fluorescent images were taken on Olympus FV1000 confocal laser scanning microscope using 40x objective.

### Western blot analysis

Cells were lysed in RIPA lysis buffer containing protease inhibitors (Roche, Indianapolis, IN) and protein concentration on cell lysates was determined according to manufacturer’s instruction using BCA Protein Assay Kit (Pierce, Rockford, IL). Twenty micrograms of proteins were loaded per well for electrophoresis after which the proteins were transferred to polyvinylidenedifluoride (PVDF) membranes and blocked with 5% milk. The blots were incubated overnight with rabbit anti-Id2 (1∶100), n-myc, p-Akt, Akt, p-FAK, FAK, p-Erk, Erk, p-Raf, Raf, p-Src, Src, p-Smad2/3, Smad2/3, integrin α5, integrin β1, integrin β3, Bcl2 and Bax (1∶1000; Cell Signaling, Danvers, MA) followed by HRP-conjugated anti-rabbit secondary antibody (1∶2000, Pierce). Blots were developed by chemiluminescence using the SuperSignal Kit (Pierce). Rabbit anti-GAPDH (1∶5000; Cell Signaling) was used as a control for protein loading variations. Densitometric analysis was performed on the western blot bands using NIH Image J software.

### Co-immunoprecipitation assay

Neuro2a AD cells were lysed in RIPA lysis buffer containing protease inhibitors. Following BCA protein analysis, 500 µg of protein per sample was first pre-cleared with1/10 volume of protein A/G agarose beads (Santa Cruz Biotechnology) at 4°C for an hour on an end-over-end rotator. After pelleting the beads by centrifugation, the supernatants were incubated overnight with 2 µg primary antibody (rabbit anti-Id2 or rabbit anti-Smad2/3) or control polyclonal rabbit IgG at 4°C on the rotator. Following overnight incubation, protein A/G agarose beads were added at 1/10 total volume and incubated for 2 hours at 4°C on the rotator. Beads were then centrifuged, washed twice with RIPA buffer and resuspended in 20 µl of 1x sample buffer (BioRad, Hercules, CA). After boiling for 5 minutes, 5 µl of the samples were loaded per well for electrophoresis and analyzed for Id2, TGFβ (1D11, R&D Systems) and Smad2/3 by Western blotting.

### Statistical analysis

Data are presented as mean ± S.D. The two-tailed Student’s t-test was used to determine statistical significance between groups unless otherwise stated. A probability level of p<0.05 was considered to be statistically significant. Statistical analysis of microarray data comparing AD and AI cells was performed based on one-way ANOVA with a 5% False Discovery Rate (FDR) criterion to correct for multiple testing.

## Results

### Neuroblastoma phenotypes have distinct gene profiles that differentiate cell types in vitro

**Figure 1:**
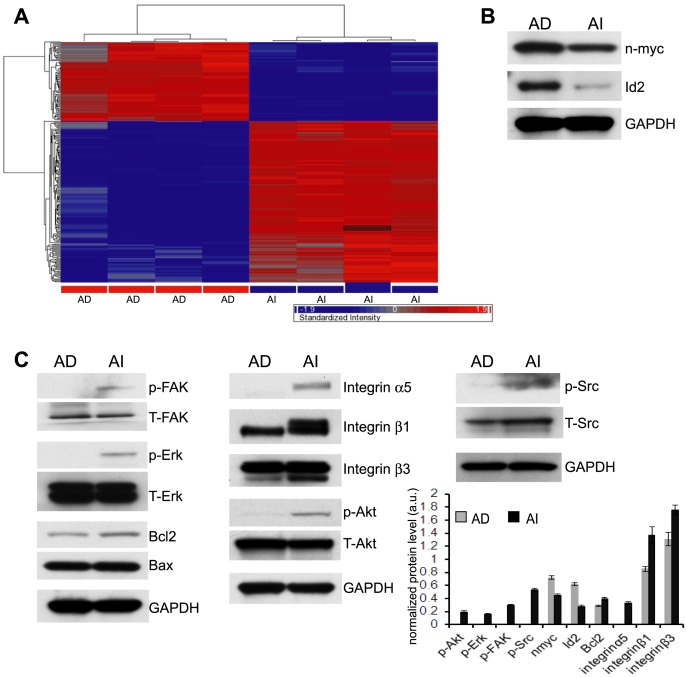
Gene expression profiling of Neuro2a cells. (**A**) Affymetrix microarray was performed with mRNA extracted from Neuro2a anchorage dependent (AD) and anchorage independent (AI) cells. 1180 differentially expressed genes (5% FDR, ≥1.5-fold change) were identified (see Table S1). In two-way hierarchical clustering analysis (clustering diagram), highly expressed genes were shown in red and weakly expressed genes in blue in AD and AI cell phenotypes (n = 4). (**B**) A representative Western blot analysis performed with protein extracted from Neuro2a AD and AI cells showed overexpression of n-myc and Id2 in the AD cells which correlated with the gene array results. (**C**) Representative Western blot analysis performed with protein extracted from Neuro2a AD and AI cells and densitometric band analysis revealed overexpression and activation of proteins involved in anoikis resistance in the AI phenotypes thereby validating the gene array profiling data. These include integrins, the anti-apoptotic protein Bcl2 and Akt, FAK/Src and Erk signaling molecules. The band intensity of the proteins was normalized to the intensity of GAPDH with the exception of phosphorylated proteins that were normalized to their total proteins. Data points represent mean ± S.D. (n = 4)

Affymetrix gene array analysis of the mouse Neuro2a cells revealed remarkable differences between the AD and AI phenotypes *in vitro* (Figure 1A). One thousand one hundred and eighty (1180) genes were differentially expressed and delineated pathways of proliferation in AD cells or tyrosine kinase activation/apoptosis inhibition in AI cells (Table S1 and Figure S1). In particular, inhibitor of differentiation 2 (Id2) was found to be 20 fold higher in AD compared to AI cells (Table S1). Id2 disrupts the anti-proliferative effects of the retinoblastoma (Rb) family tumor suppressor proteins thus allowing cell cycle progression [28]. Id2 is critical for cell proliferation and is the oncogenic effector of N-MYC in human neuroblastoma [27], [29]. Indeed, our gene array analysis revealed that n-myc gene expression was 3.5 times higher in the AD cells (Table S1). Using western blot analysis we validated that Id2 and n-myc proteins are both overexpressed in the AD phenotype of Neuro2a cells (Figure 1B). In addition, a wide array of genes involved in anoikis resistance including Bcl2, PI3K/Akt, EGFR, Ras, integrin α1, α3, α5, β1 and β3 were found to be overexpressed in the AI cells (Table S1).

Typically, cells grown under conditions of loss of cell-matrix anchorage and serum starvation undergo anoikis (apoptotic cell death following detachment). In our recent study we reported that only 35% of Neuro2a cells grown under these conditions as anchorage independent cells with EGF and bFGF undergo apoptosis [19]. The addition of growth factors (EGF and bFGF) in the culture medium may have activated growth factor receptors and other anti-apoptotic and survival pathways via PI3K, FAK, and Raf contributing to the anoikis resistance of the surviving AI cells. The Raf/MEK/Erk and PI3K/Akt signaling cascades regulate cell growth, tumorigenesis and drug resistance [30]. Since the gene array analysis exhibited increased expression of Bcl2, integrins, Ras and PI3K we sought to determine the expression pattern of the proteins of these pathways by a comprehensive Western blot analysis. We found that integrin β1, β3 and α5 were overexpressed in AI cells compared to AD cells (Figure 1C). The ratio of anti-apoptotic Bcl-2 to pro-apoptotic Bax proteins was also increased in the AI cells. Moreover, we observed that Akt, FAK/Src, and Erk pathways were all activated in AI cells as determined by the increased phosphorylated states of these proteins (Figure 1C). These findings suggest that AI cells evade anoikis by the activation of several key survival pathways. The transcriptional and translational profiles of the two phenotypes of Neuro2a cells closely correlated with their phenotypic characteristics; specifically, the highly proliferative feature of the AD cells compared to the less proliferative and anoikis resistant properties of the AI cells.

**Figure 2:**
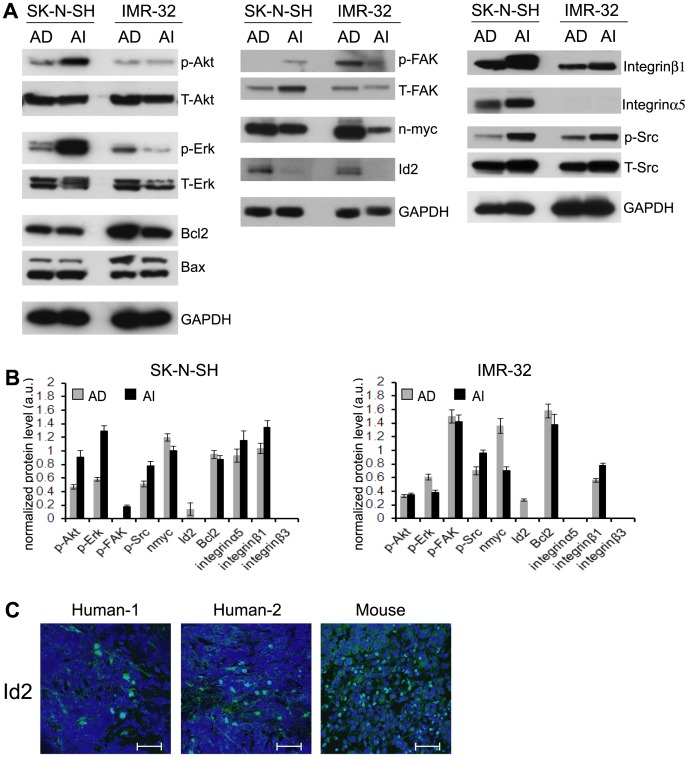
Differential protein profile in the AD and AI phenotypes of human neuroblastoma cells. (**A**) A representative Western blot analysis performed with protein extracted from the AD and AI phenotypes of SK-N-SH and IMR-32 cells showed overexpression of n-myc and Id2 in the AD phenotype of both the cell lines. AI phenotypes of SK-N-SH cells show overexpression and activation of proteins involved in anoikis resistance including integrins and Akt, FAK/Src and Erk signaling molecules. For the IMR-32 cells, the phenotypic transition did not affect these pathways to the same extent. (**B**) Quantification of protein levels by densitometric band analysis (n = 3). The band intensity of the proteins was normalized to the intensity of GAPDH with the exception of phosphorylated proteins that were normalized to their total proteins. Data points represent mean ± S.D. (n = 3) (**C**) Immunofluorescence staining reveals the expression of Id2 protein in two human neuroblastoma specimens as well as in mouse tumor (Id2: green; nucleus: blue). The images were captured using 40x objective. Scale bar: 50 µm.

We have previously reported that human neuroblastoma cell lines SK-N-SH and IMR-32 also demonstrated reversible phenotypic transformation and distinct molecular marker heterogeneity of their AD and AI cells [19]. We therefore sought to determine if the human neuroblastoma cell lines exhibit differential protein profiles similar to that observed in Neuro2a. We found that SK-N-SH cells closely resembled Neuro2a as evident from the over-activation of Akt, FAK/Src and Erk pathways in the AI cells, whereas the phenotypic transition of IMR-32 cells did not affect these pathways to the same extent (Figure 2A, B). However, Id2 and n-myc were found to be overexpressed in the AD cells of both human cell types (Figure 2A, B). Furthermore, immunofluorescence staining revealed the expression of Id2 protein in human and mouse neuroblastoma tumor specimens (Figure 2C). Taken together, these results suggest that Id2 most likely plays a significant role in neuroblastoma tumor cell growth.

### Id2 is a key regulator of cell proliferation and phenotypic transition in neuroblastoma cells

**Figure 3:**
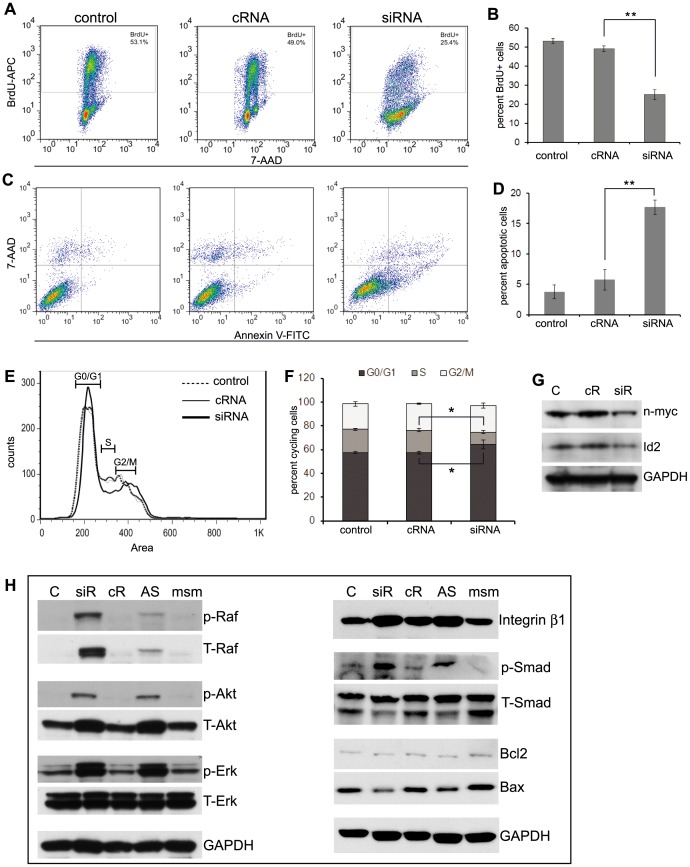
Id2 down regulation drives Neuro2a AD cells towards the AI phenotype. (**A, B**) BrdU incorporation assay demonstrated that transfection of AD cells with Id2-siRNA reduced the rate of proliferation. The apoptotic cells were excluded by gating out the sub-2n cells. (**C**, **D**) AnnexinV staining revealed increased apoptosis in AD cells following transfection with Id2-siRNA. The percent apoptotic cells in the graph represent the sum of early and late apoptosis. (**E, F**) Cell cycle analysis showed reduced number of cells entering in S-phase after Id2 inhibition. (**G**) Western blot analysis validated the decreased expression of Id2 protein after Id2 inhibition in the AD cells. (**H**) Representative bands from Western blot analysis revealed over activation of Akt, Raf, Erk and Smad pathways and overexpression of Integrin β1 protein in AD cells after Id2 down regulation indicating activation of anoikis resistant pathways. Data points represent mean ± S.D. (n = 4–6). *p<0.04 and **p<0.0001 by Student’s t-test. Control or C: AD cells; siR or siRNA: AD cells transfected with siRNA against Id2; cR or cRNA: AD cells transfected with nonsense siRNA control; AS: AD cells transfected with Id2 antisense oligonucleotide; msm: AD cells transfected with mismatched oligonucleotide.

**Figure 4:**
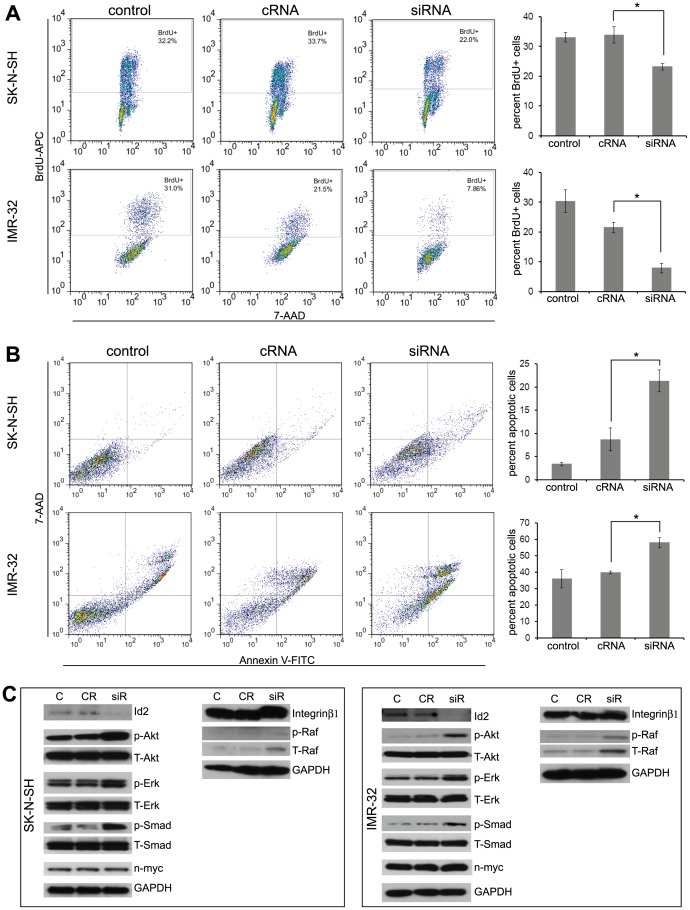
Effect of Id2 down regulation on human neuroblastoma cell lines. (**A**) BrdU incorporation assay demonstrated that transfection of AD phenotype of SK-N-SH and IMR-32 cells with Id2-siRNA reduced the rate of proliferation. The apoptotic cells were excluded by gating out the sub-2n cells. (**B**) AnnexinV staining revealed increased apoptosis in AD phenotype of SK-N-SH and IMR-32 cells following transfection with Id2-siRNA. The percent apoptotic cells in the graphs represent the sum of early and late apoptosis. (**C**) Representative bands from Western blot analysis revealed over activation of Akt, Erk and Smad pathways and overexpression of Integrin β1 protein in both SK-N-SH and IMR-32 cells after Id2 down regulation. Data points represent mean ± S.D. (n = 4–6). *p<0.004 by Student’s t-test. Control or C: AD cells; siR or siRNA: AD cells transfected with siRNA against Id2; cR or cRNA: AD cells transfected with nonsense siRNA control.

Id2 mediates mitogenic signals, inhibits differentiation and plays a critical role in cancer development and metastasis [31], [32], [33]. Due to its known function as an effector of n-myc and its remarkable differential expression in the cell phenotypes, we reasoned that Id2 could play a key role in reversible adaptive plasticity in the neuroblastoma cells. We hypothesized that the AI cells lose their proliferative potential due to a deficit in n-myc and Id2 expression under conditions in which loss of cell-matrix anchorage or serum starvation occurs. The mechanism by which loss of proliferation occurs could be through competitive binding of Rb. Diminished Id2 enables Rb binding to the transcription factor E2F, thus blocking progression into the S phase of cell cycling (free E2F induces S-phase genes) and inhibiting proliferation [34], [35], [36], [37]. Subsequently the cells either undergo apoptosis or develop anoikis resistance. To test this hypothesis, we first determined the effect of Id2 down regulation on proliferation, apoptosis and cell cycle of the AD phenotype of Neuro2a, SK-N-SH and IMR-32 cells. After transfecting AD cells with Id2-siRNA, we demonstrated decreased expression of Id2 protein by western blot analysis (Figure 3G and 4C). BrdU incorporation revealed a significant decrease in cell proliferation (Figure 3A,B and 4A) and cell cycle analysis exhibited fewer cells in the S-phase of the cell cycle (Figure 3E,F) of Id2-suppressed neuroblastoma cells compared to cells transfected with a control siRNA. In addition, we found that Id2 inhibition induced significant apoptosis in the AD phenotype of all the neuroblastoma cells studied (Figure 3C,D and 4B). Inhibition of Id2 by Id2-antisense oligonucleotide (Id2-AS) demonstrated similar results of reduced proliferation, increased cell cycle arrest and increased apoptosis compared to a mismatched oligonucleotide (Id2-msm) (Figure S2). Furthermore, western blot analysis revealed increased activation of the anoikis resistant pathways like Raf/Erk and Akt with overexpression of integrin β1 after Id2 down regulation in both mouse and human cell lines (Figure 3H and 4C). An increase in the activation of Smad pathway was also evident with Id2 inhibition (Figure 3H and 4C). These observations indicate that inhibition of Id2 in the AD cells shifts the cells towards an AI-like phenotype with decreased proliferation and up-regulation of anoikis resistant pathways.

**Figure 5:**
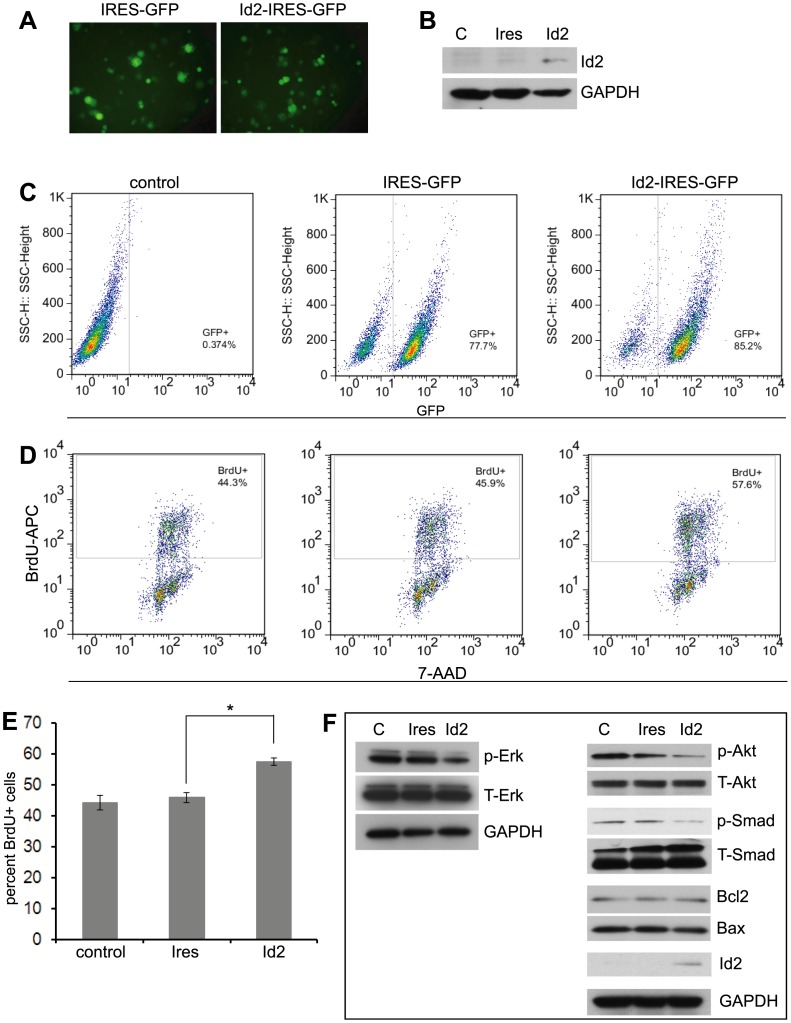
Id2 overexpression increases cell proliferation in Neuro2a AI cells. (**A**) Expression of green fluorescent protein (GFP) is evident in AI cells transfected with IRES-GFP or Id2-IRES-GFP plasmid. (**B**) Id2 protein is expressed in AI cells after Id2-IRES-GFP transfection, whereas Id2 protein was absent in AI cells transfected with IRES-GFP. (**C**) Representative plots showed 77–85% transfection efficiency. (**D, E**) BrdU incorporation assay indicating increased rate of proliferation in AI cells after Id2 overexpression. The apoptotic cells were excluded by gating out the sub-2n cells. (**F**) Representative bands from Western blot analysis revealed that overexpression of Id2 reduced the activation of Erk, Akt and Smad pathways in AI cells. Data points represent mean ± S.D. (n = 4–6). *p<0.04 by Student’s t-test. C: AI cells, Ires: AI cells transfected with IRES-GFP, Id2: AI cells transfected with Id2-IRES-GFP.

We next sought to assess the effects of forced overexpression of Id2 on the AI phenotype of Neuro2a cells by transfecting the AI cells with IRES-Id2-GFP plasmid. The transfection (Figure 5A,C) led to an increase of endogenous Id2 compared to the empty IRES-GFP plasmid (Figure 5B). Consistent with Id2 up regulation, BrdU incorporation showed an increased rate of proliferation (+40% compared to the empty plasmid, Figure 5D,E) and clearly down regulated the activation of Erk, Smad and Akt (anoikis resistant pathways) (Figure 5F).

**Figure 6:**
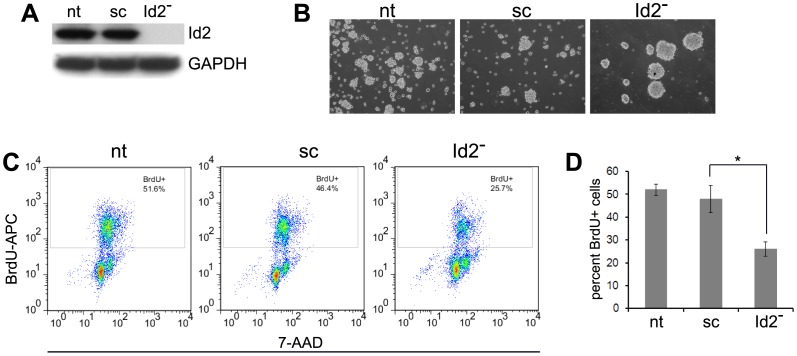
Stable knockdown of Id2 accelerates phenotypic transition. (**A**) Western blot analysis reveals complete knockdown of Id2 on Neuro2a AD cells transduced with Id2-shRNA lentiviral particles. (**B**) After only 4 days in NeuroCult complete media, the Id2 knockdown cells readily formed dense large spheres while the scrambled-shRNA transduced cells and the non-transduced AD cells formed loose smaller spheres at the same time point suggesting that Id2 knockdown accelerates the transition from AD to AI phenotype in Neuro2a cells. The images were captured on an Olympus ckx41 microscope using 10x objective. (**C, D**) BrdU incorporation assay demonstrated that transduction of Neuro2a AD cells with Id2-shRNA lentivirus reduced the rate of proliferation. The apoptotic cells were excluded by gating out the sub-2n cells. Data points represent mean ± S.D. (n = 4). *p<0.006 by Student’s t-test. nt: nontransduced Neuro2a AD cells; sc: Neuro2a AD cells transduced with scrambled shRNA lentivirus; Id2^−^: Neuro2a AD cells transduced with Id2 shRNA lentivirus.

To determine whether stable knockdown of Id2 on AD cells will expedite the phenotypic transition, we transduced the Neuro2a AD cells with Id2-ShRNA lentiviral particles. The transduced cells were tested for Id2 expression (Figure 6A) and proliferation rate. Subsequently, the cells were plated on NeuroCult complete media (AI condition) and monitored for sphere formatting ability. After only 4 days, the Id2-shRNA transduced cells formed large dense spheres in comparison to the control or scrambled shRNA transduced cells which formed small loosely bound spheres (Figure 6B). Furthermore, the Id2 knockdown cells were found to be less proliferative than the non-transduced or scrambled shRNA transduced cells (Figure 6C,D). Taken together, these results support the hypothesis that Id2 expression maintains the proliferative phenotype of AD cells and acts as a negative regulator of the phenotypic transition from AD cells to AI cells.

### Id2 prevents phenotypic transition by binding TGFβ/Smad and inhibits activation of pathways inducing anoikis resistance

**Figure 7:**
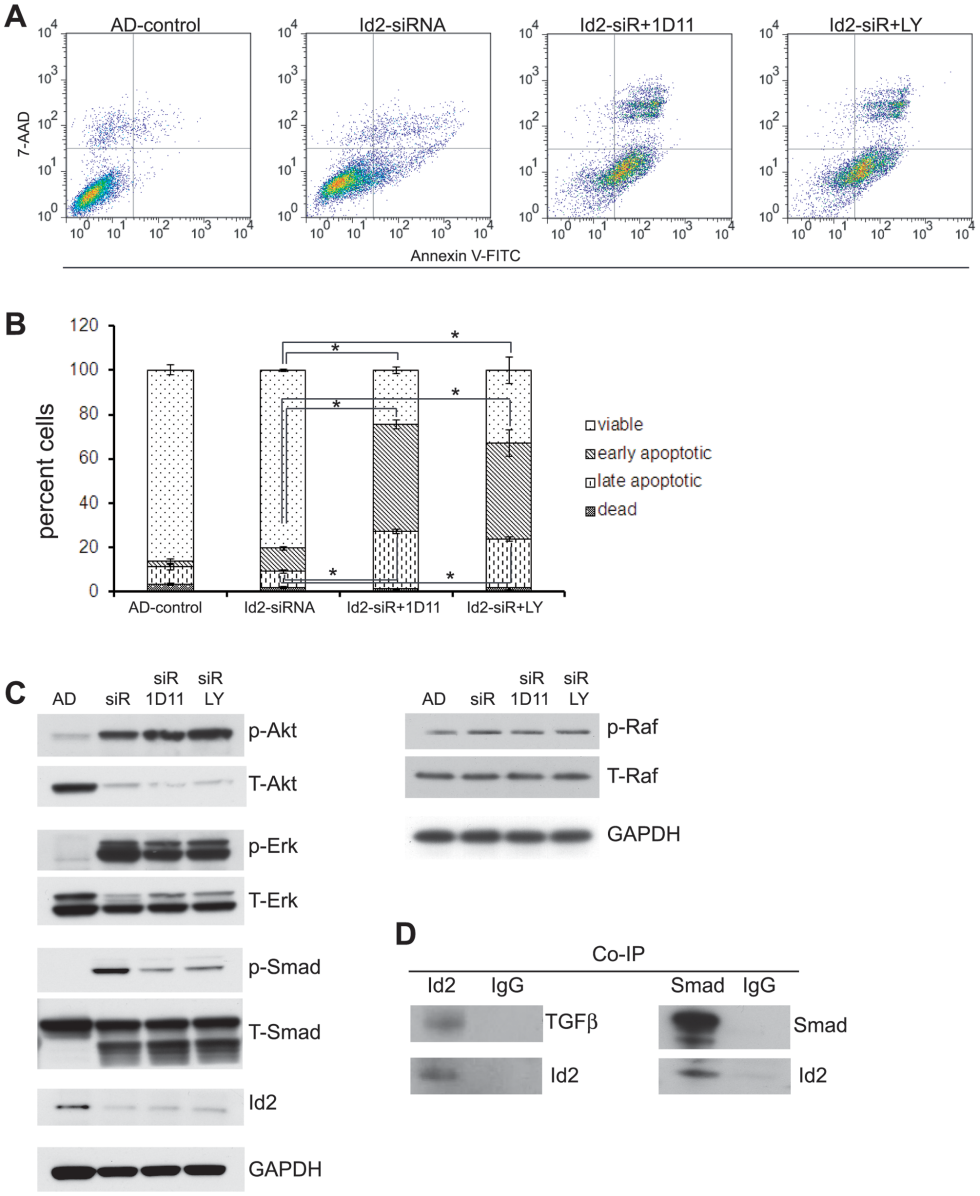
Id2 functions partially through TGFβ pathway. (**A, B**) The TGFβ neutralizing antibody (1D11) and its I/II receptor inhibitor (LY2109761) induced apoptosis in the Id2-inhibited AD cells. Data points represent mean ± S.D. (n = 3). *p<0.0001 by Student’s t-test. (**C**) Representative bands from Western blot analysis revealed decreased Smad2/3 activation in the presence of both TGFβ inhibitors with no change in Akt, Raf, Erk or Bcl2 pathways when compared to the Id2-suppressed cells. (**D**) Co-immunoprecipitation study shows Id2 binding to both TGFβ and Smad2/3.

**Figure 8:**
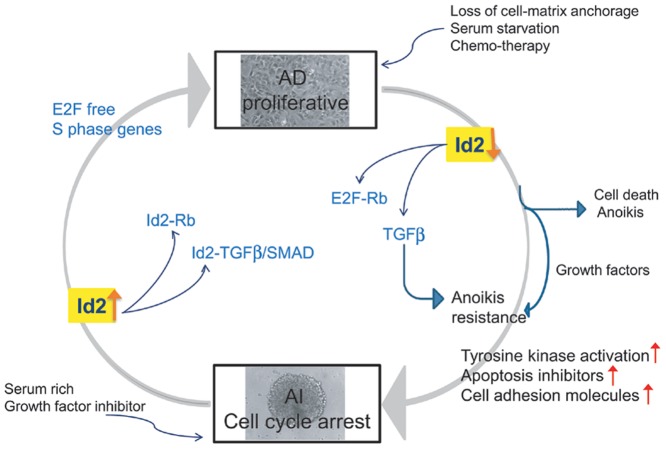
A model depicting the role of Id2 in Neuro2a phenotypic transition. We show that the AI cells lose their proliferative potential due to loss of n-myc and Id2 expression when conditions induce loss of cell-matrix anchorage or serum starvation. The mechanism by which this occurs is through competitive binding of retinoblastoma (Rb) and TGFβ. Diminished Id2 enables Rb binding to E2F, thus blocking progression into the S-phase of cell cycling (free E2F induces S-phase genes) and inhibiting proliferation. Subsequently the cells either undergo apoptosis or develop resistance to anoikis. Concurrently, inhibiting Id2 enables TGFβ to activate the pathways of anoikis resistance allowing the cells to adapt to unfavorable conditions.

Transforming growth factor β (TGFβ) induces epithelial to mesenchymal transition (EMT) in carcinoma cells and promotes tumor invasion [38], [39], [40]. Id2 is known to be a key negative regulator of TGFβ-induced EMT in epithelial cells [31], [41] suggesting that the effect of Id2 on the neuroblastoma phenotypic switch may be mediated by TGFβ. Although gene array analysis revealed increased mRNA expression of TGFβ and Smad2/3 in AD cells compared to AI cells (Table S1), western blot analysis showed no activation of the TGFβ/Smad2/3 pathway in the AD cells (Figure 7C). Therefore, we predicted that high expression of Id2 may inhibit the TGFβ function and that suppression of Id2 on AD cells would increase the TGFβ functionality by activation of Smad2/3 and also Raf/Erk pathways thereby causing the cells to overcome anoikis and become anchorage independent. Indeed, western blot analysis revealed activation of Smad2/3, Raf and Erk following Id2 down regulation (Figure 7C). Treatment of Id2-suppressed AD cells with 1D11 (TGFβ neutralizing antibody) or LY2109761 (TGFβ type I/II receptor inhibitor) induced remarkable apoptosis (+615% by 1D11 and +553% by LY2109761, see Figure 7A,B) and blocked the phosphorylation of Smad 2/3 (Figure 7C). Further evidence corroborating the suppressive effect of Id2 on the TGFβ pathway was observed in co-immunoprecipitation experiments in which Id2 bound both TGFβ and Smad2/3 (Figure 7D) suggesting an inhibitory action similar to that of Id2 on Rb. Taken together, the results show that Id2 functions as a negative regulator of TGFβ/Smad in neuroblastoma cells and activation of the TGFβ pathway is at least partially responsible for the transition of AD cells to an AI phenotype (Figure 8).

### Tumor growth in a mouse neuroblastoma model is suppressed by multi drug treatment targeting molecular mechanisms of reversible adaptive plasticity

**Figure 9:**
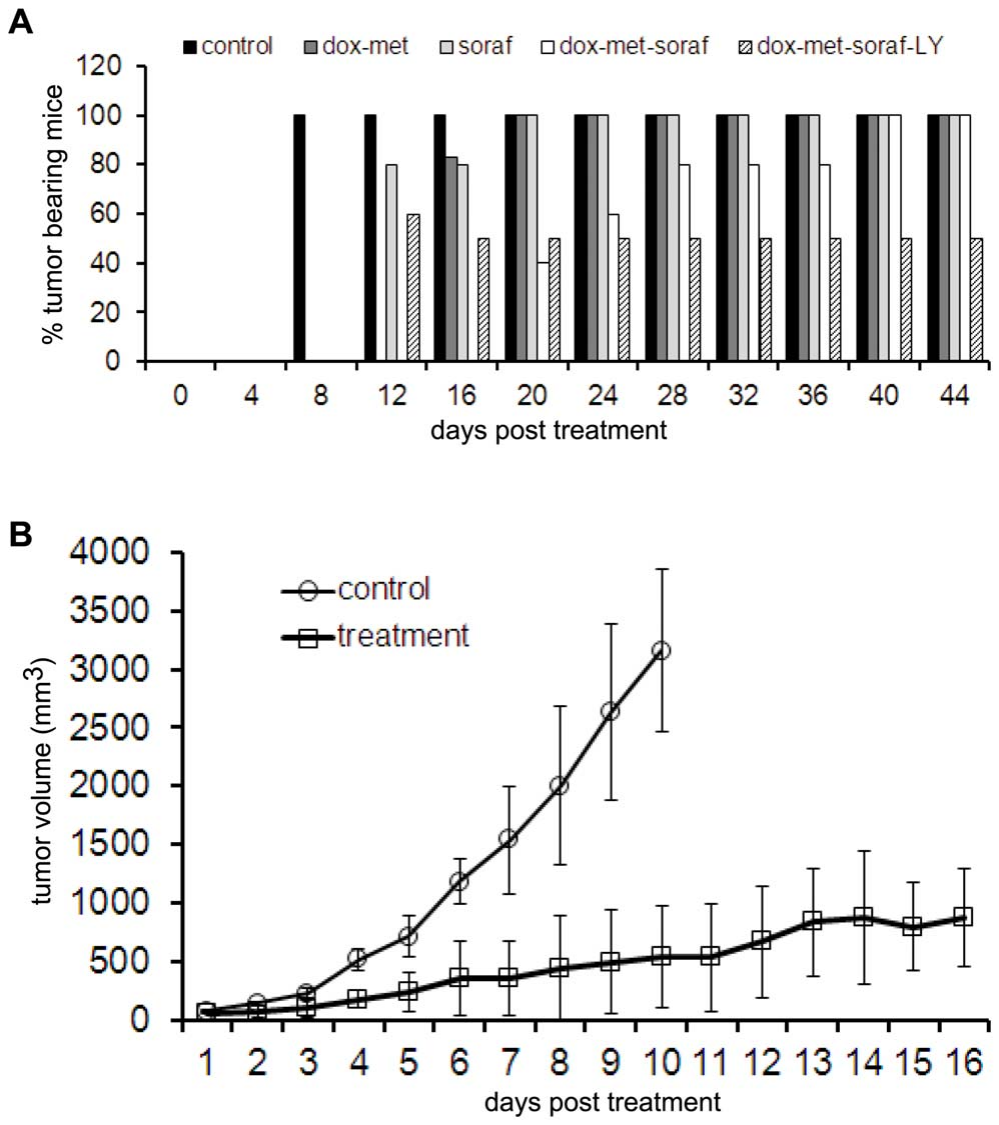
Treatment targeting AD and AI phenotypes as well as adaptive plasticity delayed mouse tumor growth. (**A**) A graphical representation of the gross tumor presence demonstrates that the combined treatment with doxorubicin, metformin, sorafenib and LY2109761 cured 50% of mice with microscopic tumors. All treatments in (A) were started one day after neuro2a inoculation (n = 10–15). (**B**) Tumor volume curve during treatment of established tumors with a combination of doxorubicin, metformin, sorafenib and LY2109761 showed remarkably suppressed tumor growth (n = 10). All mice in the control group were sacrificed by day 11 after the tumors reached 20 mm in diameter. The drug treatment in (B) was started after 5 mm tumor was visible in mice. Dox: doxorubicin, met: metformin, soraf: sorafenib, LY: LY2109761.

We have previously reported that irrespective of the phenotype (AD or AI) originally implanted in mice, neuroblastoma tumors grown *in vivo* show phenotypic heterogeneity with molecular marker signatures of both phenotypes and are indistinguishable in growth or histologic appearance. Simultaneously targeting both phenotypes with chemotherapy and growth factor inhibition slowed tumor growth in mice but promoted emergence of other variant phenotypes [19]. Since the Raf/Erk pathway is the convergence point of several other cell survival regulatory pathways that are found to be activated in AI cells, we proposed that inhibition of non-receptor tyrosine kinase signaling by the multi-kinase inhibitor sorafenib [42], [43] may represent an attractive approach to targeting the AI cells in mouse tumors. Similarly, the highly proliferative AD cells were targeted with the chemotherapy drug doxorubicin in the presence of metformin [19], [44]. We also reasoned that simultaneously targeting both phenotypes with chemotherapy and sorafenib plus the addition of a TGFβ inhibitor, LY2109761 would enhance the combinatorial effect and prevent reversible phenotypic adaptation from occurring. To test this rationale, mice were treated with reagents alone or in combination. Mice were treated with doxorubicin, metformin and sorafenib for three weeks starting a day after their tumor challenge, while mice that were treated with doxorubicin, metformin, sorafenib and LY2109761 were only treated for two weeks due to anticipated drug toxicity. In this model of neuroblastoma, we found that the combination of drugs targeting both AD and AI phenotypes together had significantly greater impact on suppressing tumor growth compared to targeting either phenotype alone (Figure 9A). When the TGFβ blocker was added to the combination to prevent phenotypic transition the effect on tumor growth was even more remarkable in which 50% of mice remained tumor free for the duration of the experiment (Figure 9A) and tumor growth was significantly suppressed (Figure 9B). These observations demonstrate the pre-clinical value of therapeutically targeting the molecular pathways governing reversible adaptive plasticity in neuroblastoma.

## Discussion

Neuroblastoma cells undergo reversible adaptive plasticity to survive unfavorable conditions and escape therapy [19]. To gain further insight into the mechanism driving this adaptation, we explored gene expression in the two heterogenous phenotypes of Neuro2a cells and examined potential target molecules involved in the phenotypic transition. We found that Id2, acting at least partially through the negative regulation of the TGFβ/Smad pathway is a key mediator of reversible adaptive plasticity. In both mouse and human neuroblastoma tumor cells, Id2 maintains the proliferative phenotype and any alteration in its expression perhaps due to microenvironmental signals may account for its phenotypic switching to a more dormant anoikis resistant phenotype (Figure 8).

Expression of Id proteins can be reactivated in human cancer and it is proposed that deregulated Id signaling may promote multiple attributes of malignant behavior [45]. The excessively high expression of Id2 in anchorage dependent cells, its function as an effector of n-myc and an oncogenic factor in neuroblastoma [27] as well as its contribution towards negative regulation of cell differentiation and positive regulation of cell cycle control [27], [32], [46] led us to investigate its role as a mediator of adaptive plasticity in neuroblastoma. Indeed, down-regulation of Id2 expression in the AD phenotype of neuroblastoma cells not only decreased proliferation and induced apoptosis but also resulted in over-activation of anoikis resistant pathways. This altered phenotype was similar to the AI cells in which anoikis resistance is evident. Conversely, overexpression of Id2 in the AI cells significantly increased their rate of proliferation. Therefore, Id2 appears critical for maintaining the proliferative AD phenotype while its suppression results in up-regulation of mechanisms governing transition to the anoikis resistant AI phenotype. These results identify and establish the distinctive functional role of Id2 in neuroblastoma tumor cell plasticity (Figure 8).

The effect of TGFβ on tissue homeostasis through the inhibition of Id proteins has been extensively studied in several cell lines including skin keratinocytes, lung epithelial cells and mammary epithelial cells [31], [47], [48] and Id2 is established as a key negative regulator of TGFβ-induced EMT in epithelial cells [31], [41]. Although in our model of neuroblastoma tumor cell plasticity, our previous findings did not detect any expression of markers indicative of EMT [19] and neuroblastoma is not a tumor of epithelial origin, it is possible that the phenotypic transition we described [19] and EMT may represent similar phenomena of tumor cell adaptation. Our findings demonstrate the critical role that Id2 plays in binding and negatively regulating TGFβ function. When Id2 is suppressed, the TGFβ pathway is activated inducing phenotypic transition to a less proliferative, anoikis resistant phenotype allowing the tumor cells to survive unfavorable or stressful conditions (Figure 8).

The Ras/Raf/Erk pathways are conserved signaling pathways that enable cells to respond to external stresses and stimuli [49], and changes in the Erk pathway can promote several effects ranging from apoptosis to exhibiting more malignant behavior depending on the cell type. In the present study, an increased activation of these pathways was noted in the AI cells as well as in AD cells following Id2-suppression. Sorafenib, competitively inhibits Raf activity resulting in attenuation of the Erk signaling pathway and is reported to have therapeutic effects in pre-clinical neuroblastoma studies [42], [43], [50]. Moreover, the anti-angiogenic effects of sorafenib in a neuroblastoma model are also described [43]. Anti-cancer efficacy of TGFβ receptor inhibitors either alone or in combination with chemotherapy is reported in animal model systems of pancreatic or breast cancers [51], [52]. In our study we observed a 50% cure rate along with decreased tumor growth and prolonged survival when LY2109761 was combined with sorafenib and doxorubicin/metformin treatment. These results demonstrate that this combination of agents significantly inhibited the growth of subcutaneous neuroblastoma tumors, most likely through targeting of the heterogenous tumor cell populations and the mechanisms governing adaptive cellular plasticity. These pre-clinical findings suggest that combined treatment strategies specifically targeting the AD and AI phenotypes as well as the pathways inducing transition may have enhanced clinical efficacy in children with neuroblastoma.

In conclusion, Id2 and TGFβ are key regulators of phenotypic transition in neuroblastoma tumor cells. Down regulation of Id2 is sufficient for the cells to change their phenotype from adherent to anchorage independent cells. This mechanistic study reveals critical pathways influencing reversible adaptive plasticity in neuroblastoma and provides the molecular and pharmacological rationale for translational therapy in patients. Our observations may also have broad implications for many other high-risk solid tumors that exhibit reversible adaptive plasticity besides neuroblastoma.

## Supporting Information

Figure S1 **Over-representation of differentially expressed genes in canonical signaling pathways of Neuro2a AD and AI cells.** The molecules with red color are up-regulated in AD cells compared to AI. The molecules with green color are down-regulated in AD cells compared to AI. Over-representation was defined as significant by a Fisher’s exact test (P<0.05). The identification of differentially expressed genes that are overrepresented in signaling pathways provides insight into molecular events that may be causally related to the gating of AD and AI phenotypes.
(TIF)

Figure S2 **Id2 down regulation in Neuro2a AD cells using anti-sense oligonucleotide.** (**a, b**) Representative plots showed that transfection of AD cells with Id2-AS (**a**) reduced the rate of proliferation as indicated by BrdU incorporation assay and (**b**) increased apoptosis. (**c, d**) Graphical representation of percentages of cells that were BrdU+ (**c**) and apoptotic (**d**) after Id2 down regulation. (**e**) Western blot analysis validated the decreased expression of Id2 protein after Id2 inhibition in the AD cells. Data points represent mean ± S.D. (n = 3). Control: AD cells, AS: AD cells transfected with Id2 antisense oligonucleotide; msm: AD cells transfected with mismatched oligonucleotide.
(TIF)

Table S1 **List of differentially expressed genes in the AD and AI phenotypes of Neuro2a cells and ingenuity pathway of gene expression.**
(XLS)
