# Supplementary figures and images for: A Mechanism Linking Id2-TGFβ Crosstalk to Reversible Adaptive Plasticity in Neuroblastoma
Source: PLoS One. 2013 Dec 23;8(12):e83521. doi: 10.1371/journal.pone.0083521 (PMC3871549; doi:10.1371/journal.pone.0083521)

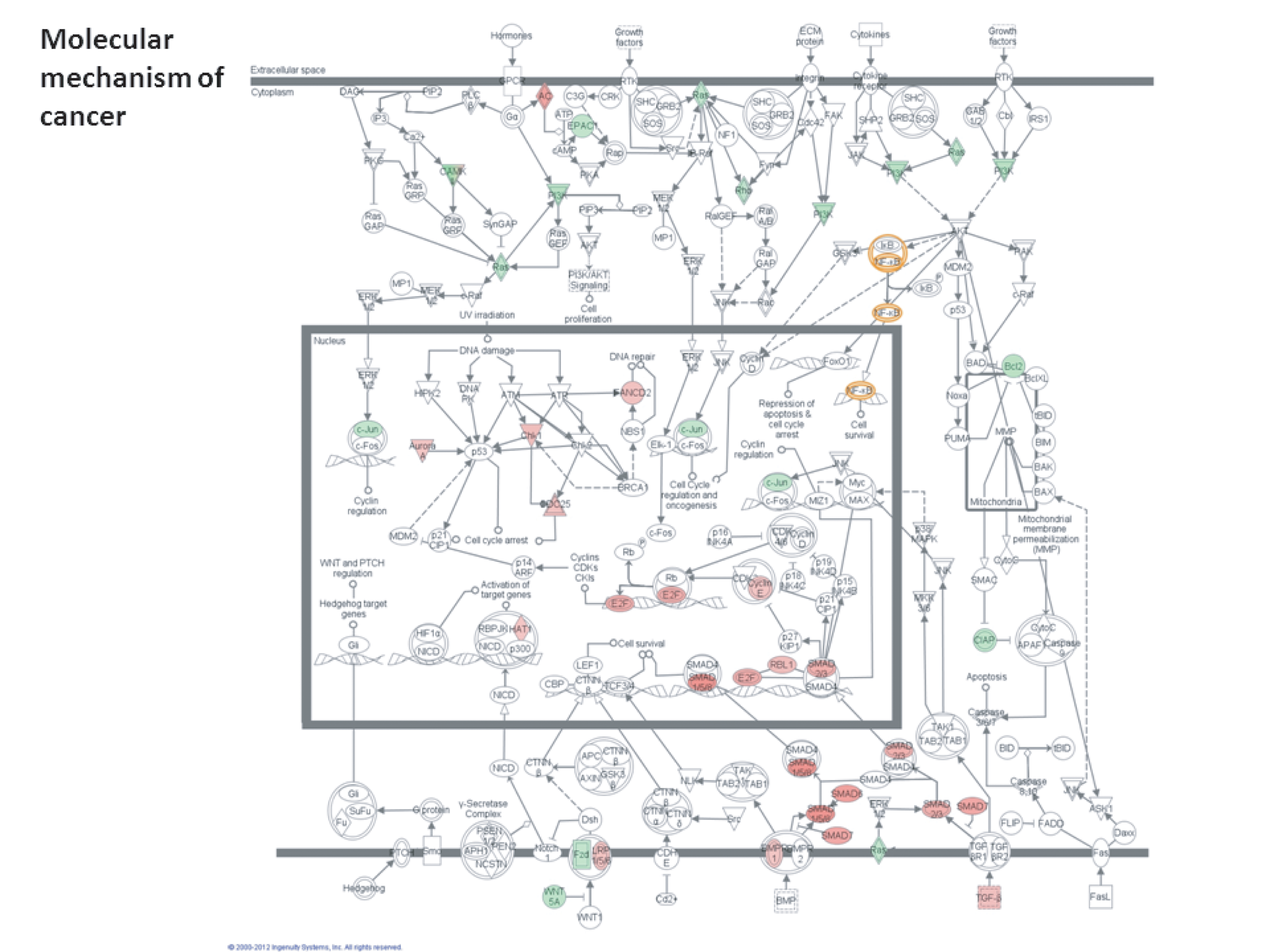

Supplement: Figure S1 — Over-representation of differentially expressed genes in canonical signaling pathways of Neuro2a AD and AI cells. The molecules with red color are up-regulated in AD cells compared to AI. The molecules with green color are down-regulated in AD cells compared to AI. Over-representation was defined as significant by a Fisher’s exact test (P<0.05). The identification of differentially expressed genes that are overrepresented in signaling pathways provides insight into molecular events that may be causally related to the gating of AD and AI phenotypes. (TIF) [file pone.0083521.s001.tif]

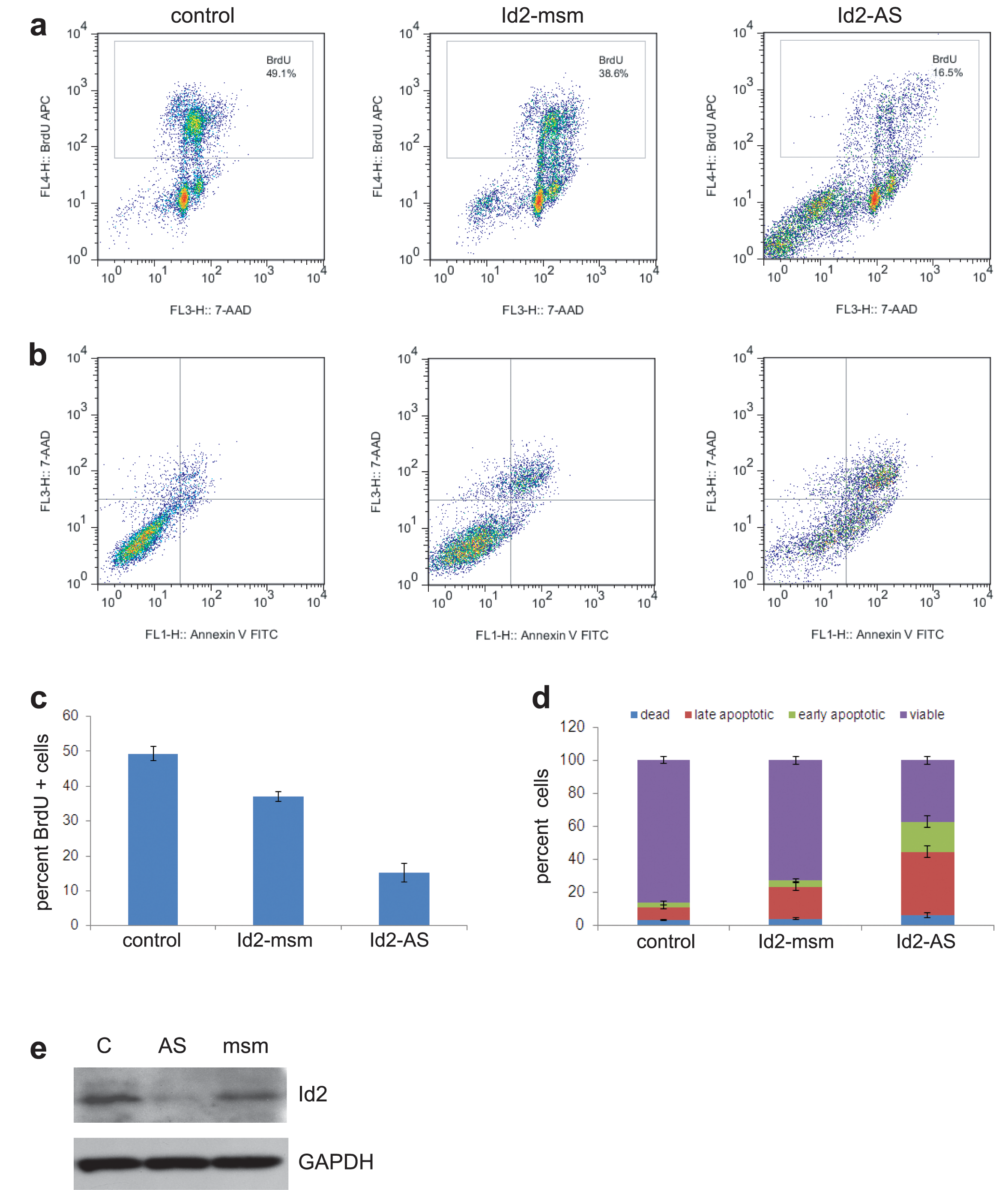

Supplement: Figure S2 — Id2 down regulation in Neuro2a AD cells using anti-sense oligonucleotide. (a, b) Representative plots showed that transfection of AD cells with Id2-AS (a) reduced the rate of proliferation as indicated by BrdU incorporation assay and (b) increased apoptosis. (c, d) Graphical representation of percentages of cells that were BrdU+ (c) and apoptotic (d) after Id2 down regulation. (e) Western blot analysis validated the decreased expression of Id2 protein after Id2 inhibition in the AD cells. Data points represent mean ± S.D. (n = 3). Control: AD cells, AS: AD cells transfected with Id2 antisense oligonucleotide; msm: AD cells transfected with mismatched oligonucleotide. (TIF) [file pone.0083521.s002.tif]
